# Effects of adding dietary bakery waste to corn on broiler growth performance, carcass traits, and feed costs

**DOI:** 10.14202/vetworld.2025.440-445

**Published:** 2025-02-19

**Authors:** Wilaiwan Sirirotjanaput, Jeerasak Chobtang, Auraiwan Isuwan, Supawadee Chimtomg, Janjira Sittiya

**Affiliations:** 1 Faculty of Animal Sciences and Agricultural Technology, Silpakorn University, Phetchaburi IT Campus, Cha-Am, Phetchaburi, 76120, Thailand; 2 Bureau of Animal Nutrition Development, Department of Livestock Development, Pathumthani, 12000, Thailand

**Keywords:** alternative feedstuffs, broiler diets, broiler performance, corn, feed cost

## Abstract

**Background and Aim:**

The rising cost of corn in livestock feed has driven interest in alternative feed ingredients. Bakery waste, a byproduct of bakery production, presents a viable substitute for corn in broiler diets. This study evaluated the effects of replacing 40% of dietary corn with bakery waste on broiler growth performance, carcass traits, and feed costs. We hypothesized that this substitution would maintain performance while reducing feed costs.

**Materials and Methods:**

A total of 240 1-day-old Ross 308 broiler chicks were randomly assigned to two dietary treatments: (T1) A control diet with 100% corn and (T2) a diet replacing 40% of corn with bakery waste. Each group had six replicates of 20 birds, housed under identical conditions with ad libitum access to feed and water for 35 days. Growth performance (feed intake [FI], body weight gain [BWG], and feed conversion ratio [FCR]) was assessed at different growth stages. Carcass traits were evaluated in selected birds, and feed cost per kilogram gain (FCG) was calculated. Statistical analysis was performed using a paired Student’s t-test, with significance set at p < 0.05.

**Results:**

No significant differences were observed in FI, BWG, or FCR between groups across all growth phases (p > 0.05). However, FCG was significantly lower in the T2 group compared to T1 (p < 0.05), indicating reduced feed costs. Carcass traits showed no major differences except for significantly lower eviscerated carcass yield and breast yield in the T2 group (p < 0.05).

**Conclusion:**

Replacing 40% of corn with bakery waste in broiler diets is a cost-effective strategy without adverse effects on growth performance. However, the reduction in breast yield suggests potential amino acid imbalances, warranting further investigation into nutrient digestibility and fat deposition. Future research should optimize bakery waste inclusion levels to ensure economic feasibility while maintaining meat quality.

## INTRODUCTION

Food waste, including household, food-processing, and restaurant waste, is a significant component of municipal solid waste. Thailand contains many important tourist attractions and is facing significant environmental challenges due to the increase in food waste from tourism and related industries. In 2021, approximately 24.98 million tons of garbage were produced in Thailand, of which 38.76% was food waste [1]. According to the United Nations Environment Program [2], the world generates more than 931 million tons of food waste (2019), representing one-third of global food production. Consequently, food waste is a global concern because it is an organic waste product that contributes to greenhouse gas emissions, which is a major form of pollution [3, 4]. Current methods for managing food waste in Thailand include resource reduction, landfilling, and recycling. As an alternative to these methods, food waste recycling for animal feed offers a potential solution for reducing food waste and selecting appropriate removal technologies.

Broiler production has increased worldwide, and production costs have also increased because of the rising cost of conventional poultry feed ingredients, such as corn [5]. Corn is one of the main energy sources for broiler rations in Thailand. A large amount of this grain is imported from other countries, which increases the cost of broiler rations. This cost is dependent on the world supply and demand of the product. Therefore, livestock producers are interested in investigating the possibility of other feed ingredients that can replace corn without negatively affecting the performance of broilers. We hypothesize that bakery waste can replace up to 40% of corn in broiler diets without adversely affecting performance while reducing feed costs. In Phetchaburi, our region, many bakery factories produce large amounts of bakery waste. The price of bakery waste is lower than that of corn because bakery waste is considered a waste product. Bakery waste, which includes bread, cookies, and other confections, is a byproduct of bakery factories. Due to its high caloric value, bakery waste can serve as a cost-effective energy source in livestock production, potentially reducing the reliance on corn and other feedstuffs [6, 7]. Bakery waste is a valuable resource, and its efficient use can also reduce the environmental impact of bakeries. The inclusion of up to 30% dried bakery waste in broiler diets has no adverse effect on the performance of chickens [6]. Yadav *et al*. [8] reported that replacing corn with bakery waste at 20%–40% in broiler diets had no negative effect on the broiler industry. In addition, the inclusion of 30% bread waste in broiler diets significantly reduced feed costs and increased profit margins [9].

Yadav *et al*. [8] have shown that including up to 40% dried bakery waste in broiler diets does not negatively impact the economic feasibility of broiler production. However, limited research has investigated the effects of bakery waste inclusion on broiler growth performance, carcass characteristics, and feed cost per gain. Therefore, we hypothesized that bakery waste can replace up to 40% of corn in broiler diets without adversely affecting performance and potentially reducing feed costs.

## MATERIALS AND METHODS

### Ethical approval

The experiment was carried out following the guidelines and rules for animal experiments at the Faculty of Animal Sciences and Agricultural Technology, Silpakorn University, Thailand (ID Project 23/2565).

### Study period and location

This study was conducted from February 2023 to July 2023 at the Faculty of Animal Sciences and Agricultural Technology Farm, Silpakorn University Phetchaburi IT Campus, Thailand.

### Preparation of dry bakery waste

The bakery waste used in this study was collected from bakery factories in Phetchaburi, Prefecture, Thailand. They consisted of bread and cookies. All samples were collected directly from the factory so, no chance of mycotoxins in the samples. The samples were oven-dried at 60°C for 24 h daily and then ground in a hammer mill (RT-34 model, Rong Tsong Precision Technology Co., Taiwan) equipped with a 250 μm screen. Powdered bakery waste was then stored in polyethylene pouches and sealed at 4°C until further use. Samples of the bakery waste were analyzed in triplicate for protein, moisture, crude fiber, ash, and ether extracts in accordance with the AOAC [10]. In addition, the gross energy of the samples was determined using a bomb calorimeter (Parr 6200 calorimeter, Parr Instrument Company, USA) (Table 1) [11].

**Table 1:** Comparison of the chemical compositions of bakery waste and corn.

Items	Chemical composition (%)					

	Dry matter	Crude protein	Ether extract	Crude fiber	Ash	Metabolizable energy (Kcal/kg)
Bakery waste	95.38	14.29	12.55	1.63	1.29	4050.00
Bakery waste^1/^	92.00	10.50	11.70	1.20	-	3862.00
Corn^1/^	89.00	8.50	3.80	2.20	-	3350.00

1/ NRC [11]

### Experimental design

A total of 240 1-day-old unsexed broiler chicks (Ross 308) were obtained from a commercial hatchery. The chicks were weighed individually and randomly divided into two groups (T1 and T2) of chicks with similar mean body weights, with each group having six replicates of 20 chickens. They were housed in litter-floored pens with rice hulls under continuous lighting and had *ad libitum* access to water for 35 days. The experimental diets used for the starter period (0–10 days), grower period (11–24 days), and finisher period (25–35 days) were mashed and formulated according to Aviagen [12]. The experimental diets included (T1) a control corn-based diet with 0% bakery waste and (T2) a diet replacing 40% of corn with bakery waste. The percentages of bakery waste in the T2 groups of starter, grower, and finisher diets were 20%, 21%, and 21%, respectively. The feed formulations were isocaloric and isonitrogenous (Table 2).

**Table 2:** Feed compositions and calculated nutrient values of experimental diets (as-fed basis).

Component	Stater (0–10 days)		Grower (11–24 days)		Finisher (25–35 days)	
		
	T1^1/^	T2	T1	T2	T1	T2
Ingredient (%)						
Corn	50.32	35.68	52.52	37.87	57.23	42.58
Soybean meal	40.07	35.72	36.87	32.52	32.09	27.74
Bakery waste	-	20.00	-	21.00	-	21.00
Soybean oil	4.69	3.64	5.86	4.81	5.96	4.90
Monocalcium phosphate	2.08	2.08	2.10	2.10	2.14	2.14
Limestone	1.29	1.27	1.29	1.28	1.31	1.30
Salt	0.51	0.32	0.46	0.27	0.46	0.27
Premi×^2/^	0.25	0.25	0.25	0.25	0.25	0.25
D, L-methionine	0.35	0.39	0.30	0.35	0.27	0.32
L-lysine HCl	0.17	0.31	0.10	0.24	0.09	0.23
L-threonine	0.12	0.19	0.08	0.14	0.05	0.12
Choline chloride	0.10	0.10	0.10	0.10	0.10	0.10
Feed cost (Baht/kg)	20.89	17.39	21.02	17.52	20.58	17.08
Nutrition value (%)						
Crude protein	23.00	23.00	21.50	21.50	19.50	19.50
Crude fat	7.03	7.89	8.23	9.09	8.44	9.31
Crude fiber	2.75	2.55	2.68	2.48	2.61	2.42
Ash	5.98	5.75	5.82	5.59	5.59	5.38
Total Ca	1.00	1.00	1.00	1.00	1.00	1.00
Total P	0.84	0.81	0.83	0.79	0.81	0.78
Metabolizable energy (kcal/kg)	3,000.00	3,000.00	3,100.00	3,100.00	3,150.00	3,150.00

1/ T1=A control diet with 100% corn, T2=A diet replacing 40% of corn with bakery waste.

2/ Premix included the following (per kg of diet): Retinol, 2.48 mg; cholecalciferol, 0.07 mg; tocopherol, 20.11 mg; menadione, 1.1 mg; thiamine, 1.4 mg; riboflavin, 5.5 mg; pyridoxine, 1.1 mg; cyanocobalamin, 12 µg; niacin, 41.3 mg; pantothenic acid, 11 mg; biotin, 41 µg; folic acid, 1.4 mg; manganese, 125 mg; iron, 282 mg; copper, 27.5 mg; zinc, 275 mg; iodine, 844 µg; selenium, 250 µg (n = 120 birds/experimental group).

### Growth performance and carcass traits

The feed intake (FI) and body weight gain (BWG) of chicks in each replicate cage were recorded during each period. The feed conversion ratio (FCR) and production efficiency factor (PEF) were calculated using the following formulas:


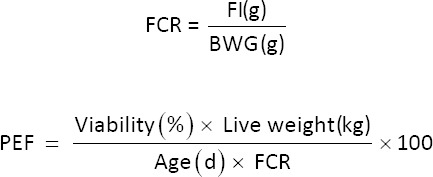


At the end of the experimental period, six birds with an average body weight (average body weight ± 100 g) from each group were used to determine carcass traits. The wings, breasts, thighs, and drumsticks were removed and individually weighed. The weights were expressed relative to the 100-g body weight.

### Cost analysis

The most recent raw ingredient prices at the time of the feed cost estimation were used. The total cost of feed was then calculated as the product of the total feed consumed over 35 days and the sum of the cost of ingredients per group. The total feed cost per kilogram of gain (FCG) of each group was calculated as the total feed cost divided by the total body weight of the birds in each group. Therefore, FCG (baht/kg) is equal to the total feed cost divided by the total body weight.

### Statistical analysis

Data on growth performance, carcass traits, and FCG were analyzed using the paired Student’s t-test to compare differences between the two dietary treatments. Results are presented as mean ± standard error. Statistical significance was set at p < 0.05. All analyses were conducted using R statistical software (version 4.0.2, R Foundation for Statistical Computing, Vienna, Austria).

## RESULTS AND DISCUSSION

The results of the proximate analysis of bakery waste and corn are presented in Table 1 [11]. According to the NRC [11], bakery waste contains more energy than corn, likely due to its higher carbohydrate content. In this study, the chemical composition of bakery waste differed from that reported by the NRC [11]. This could be attributed to differences in bakery sources, components, and processing methods; hence, it is recommended that the nutrient content of bakery waste be analyzed before it is included in animal diets. Our study revealed that the feed cost was lower when corn was replaced with bakery waste (Table 2), leading to a decrease in production cost since feeds account for 65%–75% of the total poultry production cost [13]. These results are also supported by those of other studies, which reported a decrease in feed costs for animals fed non-conventional feedstuffs in livestock diets [7, 14, 15].

**Figure 1:**
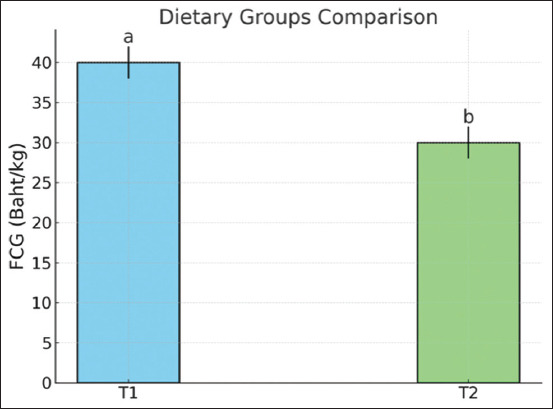
FCG of broiler chickens fed diets replacing corn with bakery waste (n = 6). FCG=The total feed cost per kilogram of gain, T1=A diet replacing 40% of corn with bakery waste, T2=A diet replacing 40% of corn with bakery waste. Different lowercase letters (a and b) indicate significant differences between groups (p < 0.05).

The effects on performance after replacing dietary corn with bakery waste are presented in Table 3. No significant differences were detected in the FI, BWG, or FCR during the starter, grower, finisher, or overall period in any group. These findings suggest that the similar metabolizable energy content of the diets across all treatments may have led to consistent FI because the chickens likely adjusted their consumption to meet their energy needs. This study is in agreement with Al-Tulaihan *et al*. [6], who reported that the inclusion of up to 30% dried bakery waste in broiler diets had no harmful effect on the performance of the birds. Sagan *et al*. [16] also reported that replacing dietary corn up to 100% with extruded Arabic bread waste did not significantly impact total BWG, total FI, or FCR at d 35. During the overall period, the FCG was lower in the T2 group than in the T1 group (p < 0.05; Figure 1), indicating that bakery waste could replace 40% of corn in the diet. The FCG was assumed to be due to the low feed cost when corn was replaced with bakery waste. The cost of corn resulted in a greater total feed cost per gain for the T1 diet compared with the T2 diet. The high cost of corn stems from its high demand and limited production. Similarly, Adeyemo *et al*. [15] reported that replacing corn with dietary biscuit waste in broiler diets decreased FCG. It can be concluded that bakery waste could be used to partially replace corn in the diets of broiler chickens to reduce feed costs and FCG without negatively affecting chicken performance.

**Table 3:** Growth performance of broiler chickens fed diets replacing corn with bakery waste at 0–35 days old (n = 120 birds/experimental group).

Items	Dietary group^1/^		p-value

	T1	T2	
Starter period (0–10 days)			
FI (g)	246.37 ± 11.61	253.82 ± 19.15	0.747
BWG (g)	132.65 ± 9.78	137.53 ± 4.06	0.660
FCR	1.89 ± 0.11	1.86 ± 0.40	0.891
Grower period (11–24 days)			
FI (g)	1079.02 ± 27.05	1055.07 ± 45.67	0.483
BWG (g)	591.08 ± 26.77	565.40 ± 25.17	0.501
FCR	1.84 ± 0.09	1.88 ± 0.07	0.732
Finisher period (25–35 days)			
FI (g)	1595.25 ± 73.15	1575.52 ± 33.78	0.812
BWG (g)	988.88 ± 36.79	984.55 ± 44.82	0.942
FCR	1.61 ± 0.06	1.61 ± 0.06	0.959
Overall period (0–35 days)			
FI (g)	2920.65 ± 90.07	2884.42 ± 43.44	0.725
BWG (g)	1712.63 ± 60.74	1687.48 ± 52.25	0.760
FCR	1.78 ± 0.07	1.78 ± 0.08	0.976
PEF^2/^	260.67 ± 28.73	267.09 ± 22.95	0.865

1/ T1=A control diet with 100% corn, T2=A diet replacing 40% of corn with bakery waste.

2/ PEF=Production efficiency factor, FI=Feed intake, BWG=Body weight gain, FCR=Feed conversion ratio

PEF is used in many countries worldwide, including Thailand, to measure the growth performance of broiler chickens [17]. As shown in Table 3, no significant differences in PEF were observed between the experimental groups during the overall study period. This could be attributed to factors involved in calculating the PEF, such as live weight, FCR, and viability, which did not differ between the groups (p > 0.05; Table 3).

In modern genetic lines, selection for rapid growth and efficient muscle protein deposition has resulted in a strong positive correlation between dietary energy, amino acid content, and breast muscle yield [18]. Likewise, diet has been shown to influence carcass weight and carcass cuts in chicken, including breast, drumstick, wings, and thighs [19]. The results of this study revealed that the carcass traits of broilers were not different between the groups, except for the significantly decreased eviscerated carcass and breast yields in the T2 group (p < 0.05; Table 4). The eviscerated carcass and breast yields are inversely related to the replacement of corn with bakery waste. Compared with corn, bakery waste contains a high fat content, leading to an increase in abdominal fat in chickens [16]. This increase is due to the reduction in the number of protein Millard reactions during the baking process, resulting in an amino acid imbalance [20, 21]. This is consistent with Carlos *et al*. [22], who reported that an imbalance of amino acids leads to fat deposition, which may result from a decrease in lysine digestibility. Additional evidence of carcass traits has been observed in several studies and has indicated that the accumulation of high fat contents in the carcasses of broiler chickens usually results in the production of low-fat breast meat [23]. This phenomenon negatively affects both producers and consumers, resulting in a reduction in carcass yield and an increase in fat accumulation. Nevertheless, incorporating bakery waste into broiler diets can benefit end producers by reducing feed costs and, consequently, the total cost of meat production. Sagan *et al*. [16] found that broilers fed a diet containing extruded Arabic bread waste exhibited a reduction in breast weight and an increase in the percentage of abdominal fat. This finding may explain the decreased carcass and breast yield of broilers fed bakery waste.

**Table 4:** Carcass traits in broiler chickens fed diets that replaced corn with bakery waste (n = 6).

Items	Dietary group^1/^		p-value

	T1	T2	
Live body weight (g)	1894.44 ± 57.43	1811.11 ± 67.10	0.352
Carcass (g/100 g BW)			
Eviscerated carcass	70.38 ± 0.64^a^	67.86 ± 0.69^b^	0.023
Thigh and drumstick	22.27 ± 0.22	22.10 ± 0.41	0.705
Breast	24.52 ± 0.60^a^	22.38 ± 0.49^b^	0.020
Wing	8.83 ± 0.12	8.78 ± 0.18	0.712

^a,b^Means within a row with different letters differ significantly at p *<* 0.05.

1/ T1=A control diet with 100% corn, T2=A diet replacing 40% of corn with bakery waste. BW=Body weight

## CONCLUSION

This study demonstrated that replacing up to 40% of dietary corn with bakery waste in broiler diets is a cost-effective strategy that does not negatively affect growth performance. Key findings indicate that FI, BWG, and FCR remained statistically similar between treatment groups (p > 0.05), confirming that bakery waste can serve as an energy-rich alternative to corn. In addition, FCG was significantly reduced in the bakery waste-fed group (p < 0.05), highlighting the economic advantage of this approach.

However, carcass evaluation revealed that while overall carcass traits remained unaffected, eviscerated carcass yield and breast yield were significantly lower in broilers fed the bakery waste diet (p < 0.05). This suggests potential nutritional imbalances, particularly in amino acid composition, which may have influenced muscle deposition and fat distribution.

The study’s strengths include its well-structured experimental design, use of industry-relevant broiler strains (Ross 308), and practical implementation of bakery waste as a feed alternative. The findings offer direct economic benefits to poultry farmers, supporting more sustainable feed formulation strategies. However, a limitation of the study is the lack of detailed nutrient digestibility analysis and amino acid profiling of bakery waste, which could have provided deeper insights into the observed reductions in breast yield. In addition, the study was short-term (35 days) and did not assess long-term effects on meat quality, fat deposition, or bird health.

Future research should focus on optimizing bakery waste inclusion levels to maintain carcass yield while maximizing cost benefits, as well as amino acid supplementation strategies to counteract potential imbalances affecting muscle deposition. Further studies on nutrient digestibility and metabolism are needed to understand how bakery waste impacts protein utilization and fat accumulation. Long-term feeding trials should be conducted to assess the effects on meat quality, fatty acid composition, and overall broiler health. In addition, environmental impact assessments should be performed to evaluate the role of bakery waste utilization in reducing food waste and promoting sustainability in livestock production. By addressing these areas, bakery waste could be further refined into a viable, cost-efficient, and sustainable feed ingredient for the poultry industry.

## AUTHORS’ CONTRIBUTIONS

WS: Performed cost and statistical analyses. JC and AI: Coordinated the research and provided guidance on the study. SC: Prepared dry bakery waste and conducted feed analyses. JS: Designed the study, conducted the field study, and drafted and edited the manuscript. All authors have read and approved the final manuscript.
